# ^1^H NMR-Based Metabonomic Study of Functional Dyspepsia in Stressed Rats Treated with Chinese Medicine Weikangning

**DOI:** 10.1155/2017/4039425

**Published:** 2017-09-28

**Authors:** Yu Guo, Zhongfeng Li, Xinfeng Liu, Xiaolan Su, Yijie Li, Jiajie Zhu, Yilin Song, Ping Zhang, Jiande D. Z. Chen, Ruhan Wei, Jianqin Yang, Wei Wei

**Affiliations:** ^1^Department of Gastroenterology, Wangjing Hospital of China Academy of Chinese Medical Sciences, Huajiadi Street, Chaoyang District, Beijing 10102, China; ^2^Beijing University of Chinese Medicine, 11 North Third Ring Road East Road, Chaoyang District, Beijing 10029, China; ^3^Department of Chemistry, Capital Normal University, 105 West Third Ring Road North Road, Haidian District, Beijing 100048, China; ^4^Division of Gastroenterology and Hepatology, Johns Hopkins Medicine, Baltimore, MD 21224, USA; ^5^Department of Chemistry, College of Sciences and Health Professions, Cleveland State University, Cleveland, OH 44115, USA

## Abstract

^1^H NMR-based metabolic profiling combined with multivariate data analysis was used to explore the metabolic phenotype of functional dyspepsia (FD) in stressed rats and evaluate the intervention effects of the Chinese medicine Weikangning (WKN). After a 7-day period of model establishment, a 14-day drug administration schedule was conducted in a WKN-treated group of rats, with the model and normal control groups serving as negative controls. Based on ^1^H NMR spectra of urine and serum from rats, PCA, PLS-DA, and OPLS-DA were performed to identify changing metabolic profiles. According to the key metabolites determined by OPLS-DA, alterations in energy metabolism, stress-related metabolism, and gut microbiota were found in FD model rats after stress stimulation, and these alterations were restored to normal after WKN administration. This study may provide new insights into the relationship between FD and psychological stress and assist in research into the metabolic mechanisms involved in Chinese medicine.

## 1. Introduction

Functional dyspepsia (FD) is one of the most common functional gastrointestinal disorders (FGIDs). It is defined according to the Rome III criteria as consisting of several upper gastrointestinal symptoms including epigastric pain or discomfort, postprandial fullness, and early satiety, lasting for 6 months at least, without any evidence of structural diseases that can explain symptoms [1, 2]. The global prevalence of uninvestigated dyspepsia (UD) is 20.8% and the overall pooled prevalence of FD diagnosed by Rome III criteria is in the range of 4.6–11.3% [3]. The nature of FD is chronic and fluctuating, and only 50% of patient with this disease experience a resolution of symptoms, whereas 15–20% of patients' symptoms are persistent [4] which lowers the quality of life and overall health, also increasing psychological distress. This condition also poses a substantial economic burden for patients and society, and costs associated with FD in the United State were as high as $18.4 billion in 2009 [5]. However, because symptoms of FD are nonspecific and the related pathophysiology is diverse, conventional treatment is often unsatisfactory and nearly 50% of patients seek out other therapies, such as complementary and alternative medicine (CAM) [6, 7].

Chinese medicine Weikangning (WKN) is derived from two Chinese classic herbal formulas, Banxia Xiexin decoction and Xiaoyaosan. The composition of WKN includes the following fourteen Chinese herbs in total:* Scutellaria Baicalensis*,* Rhizoma Zingiberis*,* Codonopsis Pilosula*,* Curcuma Aromatica*,* Magnolia Officinalis*,* Radix Paeoniae Alba*,* Rhizoma Corydalis*,* Radix Bupleuri*,* Rheum Officinale*,* Rhizome Pinelliae Preparata*,* Rhizoma Coptidis*,* Fructus Amomi*,* Glycyrrhiza Uralensis,* and* Fructus Jujubae*. Based on a meta-analysis of randomized controlled trials comparing Chinese medicines to Western medicines, Banxia Xiexin decoction showed better clinical therapeutic effect in the treatment of FD [8]. It has also been reported that Xiaoyaosan has a strong antidepressant effect in treating chronic unpredictable mild stress (CUMS) depression both in plasma and urine metabonomic studies [9, 10]. In our previous research which considered the association between psychological factors and FGIDs [11], we adopted the method of stimulating rats via tail damping to established an FD model [12]. Further, we revealed the antioxidant effect of WKN partially explained pharmaceutical mechanisms against FD in the result of proteomic analysis [13].

As a branch of system biology, metabonomics applies a “top-down” strategy to offer a practical approach to reflect the metabolic terminal changes that relate directly to integrated system activity and metabolic network that are influenced by both host genetic and environmental factors [14, 15]. Corresponding with the holistic concept of Chinese medicine, metabonomics provides a comprehensive, systems-biology method to uncover the pharmacodynamic actions of Chinese herbal medicine and explore potential biomarkers for efficacy evaluation [16]. High-resolution NMR spectroscopy, a commonly used multiparametric metabolic profiling technology, can provide rapid, quantitative, and nondestructive analytical methods with high reproducibility and repeatability [15, 17]. An NMR-based approach of metabonomics combined with multivariate statistical analytical methods has previously been applied successfully in the field of gastroenterology [18, 19].

However, there is still a lack of metabonomic study of FD in a stressed rat model with the application of NMR spectroscopy at present. In this study, the method of NMR-based metabonomics was used to screen and identify the changes of endogenous metabolites in stressed rat serum and urine. The primary goal of the present work was to determine the potential biomarkers of FD stressed model and explore the intervention mechanism of Chinese medicine WKN against FD by NMR.

## 2. Materials and Methods

### 2.1. Preparation of Weikangning

*Scutellaria Baicalensis* (90 g),* Rhizoma Zingiberis* (90 g),* Codonopsis Pilosula* (150 g),* Curcuma Aromatica* (180 g),* Magnolia Officinalis* (100 g),* Radix Paeoniae Alba* (150 g),* Rhizoma Corydalis* (100 g),* Radix Bupleuri* (90 g),* Rheum Officinale* (60 g),* Rhizome Pinelliae Preparata* (100 g),* Rhizoma Coptidis* (80 g),* Fructus Amomi* (80 g),* Glycyrrhiza Uralensis* (90 g), and* Fructus Jujubae* (90 g), all constituents of the Chinese medicine WKN (ten times of the clinical dosage), were purchased from Beijing Tongrentang Yinpian Co., Ltd. (Bozhou, China). All raw materials of WKN were mixed and extracted twice with boiling water, each time for 1.0 h, dried in vacuo (70°C), and ground into powder form (375 g) for use. Before drug administration, the powder was dissolved in purified water with a volume of 15 ml/kg (rat body weight) at a dose of 3.375 g/kg, which is determined by the surface area ratio between experimental animals and human body based on the clinical dosage of WKN. WKN was authenticated and prepared by Professor Jian Ni, Beijing University of Chinese Medicine.

### 2.2. HPLC Chromatograms of Weikangning

All analyses were performed on a Shimadzu HPLC system, equipped with LC-20AT pump, a Shimadzu SCL-10A system controller, and a SPD-20A DAD-UV detector. The HPLC analysis was performed on an Agilent Eclipse XDB-C_18_ column (250 mm × 4.6 mm, 5 *μ*m) with a constant rate of 1.0 ml/min at 30°C. The mobile phase consisted of 0.2% (v/v) phosphoric acid in water (A) and acetonitrile (B). The following gradient elution was used: 0–5 min, 12% B; 5–10 min, 12 → 20% B; 10–20 min, 20 → 25% B; 20–25 min, 25 → 30% B; 25–30 min, 30 → 65% B; 30–40 min, 65 → 90% B. The injection volume was 10 ml. The wavelength was set at 275 nm for quantitative analysis. Reference standards of baicalin (batch number 110715-201016, purity > 98.0%) and berberine hydrochloride (batch number 110713-201212, purity > 98.0%) were purchased from National Institutes for Food and Drug Control (Beijing, China). Methanol (HPLC-grade) was obtained from Fisher (USA). HPLC-quality water was obtained using a Cascada IXwater Purification System (Pall Co., USA).

### 2.3. Animal Handling and Drug Administration

Animal experiments were conducted in accordance with the Guidelines for Animal Experimentation of Beijing University of Chinese Medicine, and the protocol was approved by the Animal Ethics Committee of the Institution. Thirty male Wistar rats (200 ± 20 g) were provided by Beijing Weitonglihua Laboratory Animal Technology Co., Ltd. (Beijing, China). All rats were maintained in standard animal conditions with regulated temperature (23 ± 2°C), humidity (60 ± 5%), and a 12 h/12 h light/dark cycle. Animals were allowed free access to food and water throughout the study period. Food and water intake were recorded daily, whereas body weights were noted down thrice a week. All animals were acclimated to the conditions for five days and individual metabolism cages for 4 hours before any experimentation.

A total of 30 rats were divided into two groups randomly, 20 for the FD model group (MS group) and 10 for the normal control group (NS group). In the FD model we adopted the method of Guo [12] of stimulating rats via tail damping, which was has been widely used in China and verified in our previous work [13]. After a 7-day modeling establishment, half of the rats in the MS group were selected randomly and assigned to the WKN-treated group (WKN group). Then, the WKN group was treated with the Chinese medicine WKN at a dose of 3.375 g/kg, which is calculated by the surface area ratio between experimental animals and human body according to the clinical dosage of WKN, whereas the MS and NS groups were given purified water. All rats were administered with drugs or purified water via gastric intubation twice a day for 14 days with a volume of 15 ml/kg (rat body weight). Daily food and water intake and stomach histopathological examination were used to assess the FD model.

### 2.4. Sample Collection and Preparation

Urine samples were collected from PM 18:00 to PM 22:00 following a 4-hour fasting period in individual metabolism cages on the predose days −1, 4, and 7 and postdose days 4, 7, 11, and 14. Centrifugation at 4000 rpm, 4°C for 10 minutes, was carried out to remove residue, and the urine samples were stored immediately at −80°C until the NMR spectroscopic analysis. Urine samples were thawed only once and prepared by mixing 550 *μ*l of urine with 55 *μ*l of 1.5 mol/L deuterated phosphate buffer (NaH_2_PO_4_ and Na_2_HPO_4_ including 0.1% TSP (sodium 3-(trimethylsilyl) propionate-2, 2, 3, 3-d4), pH 7.47), adding D_2_O up to 550 *μ*l when the urine volume was insufficient. After standing at room temperature for 5 minutes, the urine-buffer mixture was centrifuged at 10,000 rpm at 4°C for 10 minutes and the supernatant (550 *μ*l) was transferred into a 5-mm NMR tube [20]. TSP served as a chemical shift reference (*δ*0.0) with a lock signal provided by D_2_O.

After injection of 10% chloral hydrate solution intraperitoneally for anesthesia (0.4 ml/100 g), blood samples were collected from abdominal aorta in the morning of the postdose day 15 after fasting for 12 hours. Following centrifugation at 4000 rpm, 4°C for 10 minutes, serum samples were kept at −80°C until metabonomic analysis. Serum samples were thawed and prepared by mixing 200 *μ*l of serum with 400 *μ*l of 45 mmol/L phosphate buffer (NaH_2_PO_4_ and Na_2_HPO_4_, pH 7.4) in 0.9% saline solution (50% D_2_O/H_2_O v/v), adding deuterated saline up to 200 *μ*l if the serum volume was insufficient. The serum-buffer mixture was kept at room temperature for 5 minutes and centrifuged at 10,000 rpm at 4°C for 10 minutes, and the supernatant (550 *μ*l) was transferred into a 5-mm NMR tube. Lactate served as a chemical shift reference (*δ*1.336).

In addition, after the FD model was established, one rat from the NS group and two rats from the MS group were selected randomly and sacrificed following anesthesia (0.4 ml/100 g) with 10% chloral hydrate solution. The stomachs were excised immediately and immersed in 10% formalin solution for histopathological examination. Then formalin-fixed stomach slices were embedded in paraffin wax, sectioned at 5 *μ*m thickness, and stained with hematoxylin and eosin (H + E) for light microscopic observation.

### 2.5. ^1^H NMR Spectroscopy Measurement

Samples were analyzed at 298 K using a VARIAN VNMRS 600 MHz NMR spectrometer (Varian Inc., Palo Alto, Calif) operating at 599.817 MHz using a 5-mm inverse-proton (HX) triple resonance probe with a *z*-axis gradient coil. One-dimensional spectra for the ^1^H NMR spectra standard sequence of the urine samples were obtained using a first increment of NOESY pulse sequence (RD-90°-*t*_1_-90°-*t*_*m*_-90°-ACQ) with water suppression obtained with irradiation on the water peak during the relaxation delay (RD = 2.0 s). The mixing time (*t*_*m*_) was 100 ms and *t*_1_ was 4 *μ*s_1_. The 90° pulse length was corrected to approximately 10 *μ*s, and at the same time, 128 transients were gathered into 64 K data points based on a spectral width of 20 ppm for each spectrum. The free induction decay (FIDs) was weighted by an exponential line-broadening factor of 0.5 Hz prior to Fourier transformation (FT). The ^1^H NMR spectra standard sequence of serum samples was water-suppressed into one-dimensional Carr-Purcell-Meiboom-Gill (CPMG) pulse sequence (RD-90°-(*τ*-180°-*τ*) n-ACQ) with a fixed total spin–spin relaxation delay 2n*τ* of 320 ms applied to weaken the broad signals of NMR from slowly tumbling molecules and preserve those from low-molecular weight compounds with some lipid components. In the collection of 64 K data points based on a spectral width of 12000 Hz and 128 scans, the FIDs are zero-filled to double size and multiplied by an exponential function with a 0.5-Hz line-broadening factor before FT.

All phase and baseline distortions of ^1^H NMR spectra were corrected manually in phase and baseline using MestReNova 7.1.0 (Mestrelab Research, Spain). The spectral region *δ*10.0–0.5 for each sample was automatically data-reduced to 1900 integral segments of equal length (0.005 ppm), whereas the regions of water resonance (*δ*5.20–4.70) were removed to weaken baseline effects. The area under the spectrum was calculated in each segmented region, which was expressed as an integral datum, and was normalized to reduce any significant concentration variation between samples. The data were exported as text files for further statistical analysis [21].

### 2.6. Statistical Analysis

The results were imported into SIMCA-P + 12.0 (Umetrics, Sweden) for multivariate statistical analysis. An unsupervised principal component analysis (PCA) model was firstly used to identify general trends and outliers through a mean-centered approach and the plotting of principal component (PC) score plots. To minimize biological analytical variation and improve the separation between groups, partial least-squares projection to latent structures-discriminant analysis (PLS-DA) and orthogonal PLS-DA (OPLS-DA) were performed for model analysis in a unit variance-scaled approach. *R*^2^ and *Q*^2^ values were used to assess the amount of variation represented by PC and to verify the robustness of the models, respectively, and the models were cross-validated by permutation tests (permutation numbers 200) [22, 23]. Variable importance in projection (VIP) values and correlation coefficients (*P*_corr_) were both used to select the metabolites with statistically significant differences. In this study the |*P*_corr_| values > 0.60207 (for degree of freedom = 9) in the modeling stage or > 0.63190 (for degree of freedom = 8) in drug intervention and VIP values > 1 were a priori considered as the cutoff value according to the literature [23]. Besides, Chenomx NMR Suite 7.51 software (Chenomx Inc., Canada) was adopted in the work of assignments of metabolites [24]. In addition, Independent-Samples* t*-test (including *t*′-test) and Mann–Whitney* U* test were used to detect significant differences in selected signals between every two groups using SPSS Statistics Base 18.0 (SPSS Inc., USA), and *P* values of less than 0.05 were considered to represent significant differences.

## 3. Results

### 3.1. Chromatograms of Weikangning Extract and Chemical Constituent Identification

The HPLC chromatograms (supplied in the Electronic supplementary information) of WKN and representative chemical constituents,* baicalin* and* berberine hydrochloride*, are shown in Figure S1 in Supplementary Material available online at https://doi.org/10.1155/2017/4039425. The result suggested the quality of WKN was controlled and the extraction method was stable and reliable.

### 3.2. Model Assessment

After 7-day stimulation via tail damping, the rats in the MS group appeared lethargic, irritable, and anxious. Daily food and water intake were significantly decreased in the MS group compared to those in the NS group with *P* = 0.001 < 0.01 and *P* = 0.000 < 0.01, respectively, and body weights of the rats in the MS and the NS group also had significant differences with *P* = 0.001 < 0.01 (Figure S2). After 14-day drug administration, the differences in daily food and water intake between the MS and the NS group were still significant (*P* = 0.043 < 0.05, *P* = 0.019 < 0.05), whereas there were no significant differences in daily food and water intake between the WKN and the NS group (Figure S3).

Microscopy examination displayed the section of gastric antrum from the MS and NS groups, presented in Figure S4. There were no signs of apparent abnormality observed in FD model stomach tissue. Based on the results of daily food and water intake and stomach histopathological examination, the experimental model was successfully established.

### 3.3. ^1^H NMR Spectra of Urine Samples

**Figure 1:**
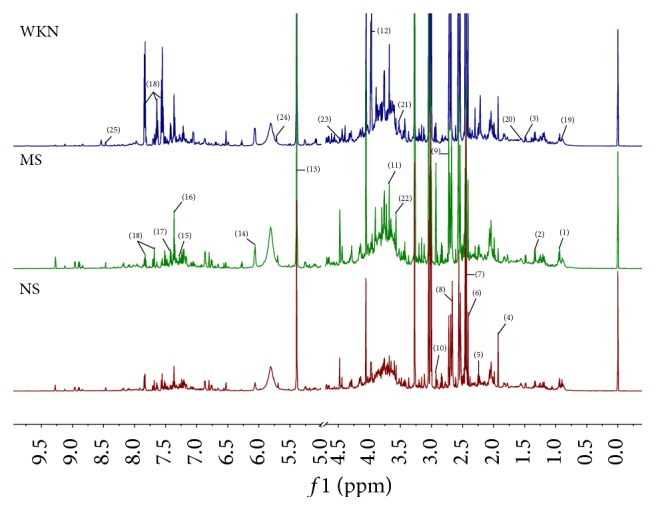
Representative 600 MHz ^1^H NMR spectra of urine samples from model group (MS), normal control group (NS), and Weikangning-treated group (WKN). Distinguished metabolites: (1) leucine, (2) lactate, (3) alanine, (4) acetate, (5) N-acetylglutamate, (6) succinate, (7) 2-oxoglutarate, (8) citrate, (9) dimethylamine, (10) trimethylamine, (11) phenylacetylglycine, (12) creatine, (13) allantoin, (14) cytidine, (15) tyrosine, (16) imidazole, (17) phenylalanine, (18) hippurate, (19) 2-hydroxyvalerate, (20) adipate, (21) glucose, (22) glycine, (23) trigonelline, (24) cis-aconitate, and (25) formate.

Representative ^1^H NMR spectra of urine obtained from the MS, NS, and WKN groups are shown in Figure 1. Endogenous metabolite assignments were based on chemical shifts according to reported literatures [25–27]. A number of metabolites were identified in the urine, including amino acids such as leucine, alanine, phenylalanine, glycine, and creatine; organic acids such as lactate, acetate, succinate, 2-oxoglutarate, citrate, hippurate, 2-hydroxyvalerate, and cis-aconitate; waste metabolites such as formate, allantoin, dimethylamine, and trimethylamine; and glucose.

### 3.4. Metabonomic Analysis of Urine Samples

**Figure 2:**
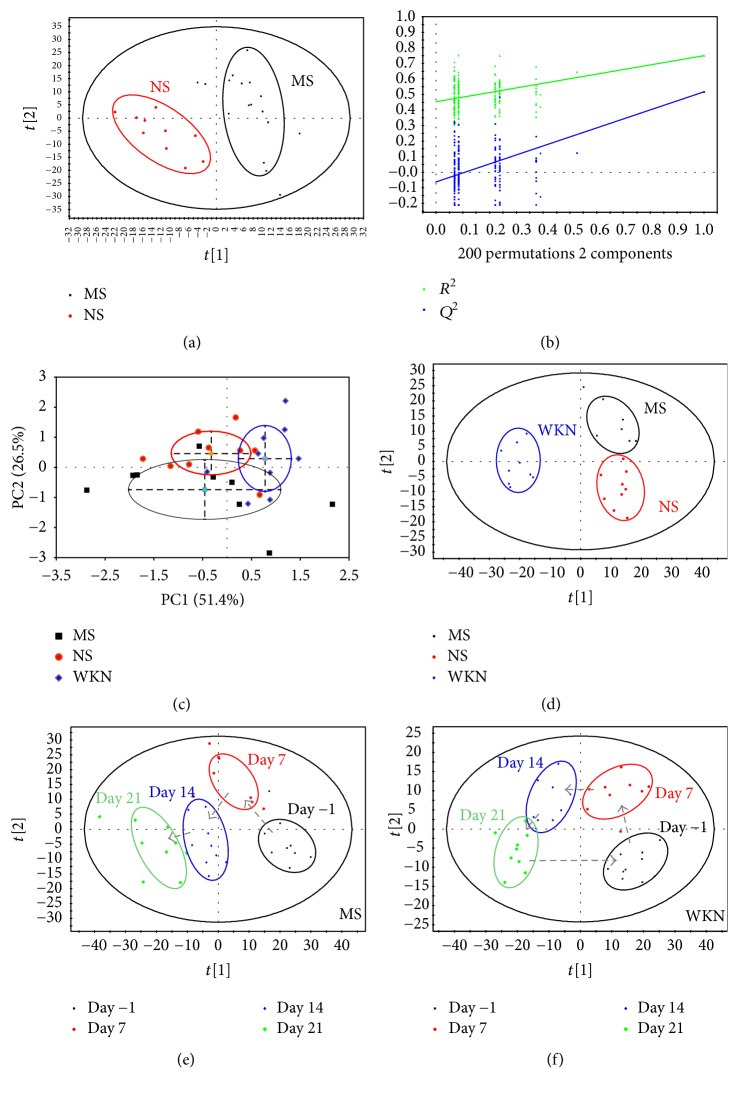
(a) PLS-DA score plot of model group (MS) and normal control group (NS) after 7-day model establishment, showing the degree of separation of the model between MS (black squares) and NS (red dots) (*R*^2^*X* = 51.1%, *R*^2^*Y* = 74.9%, *Q*^2^ = 51.7%); (b) a 200-iteration permutation test in the above PLS-DA model showing *R*^2^ (green triangles) and *Q*^2^ (blue squares) values from permuted analysis (bottom left) significantly lower than the corresponding unpermuted values (top right); (c) PCA score plot of MS, NS, and Weikangning-treated group (WKN) after 14-day drug treatment, performing discrimination between MS (black squares), NS (red dots), and WKN (blue diamonds) (*R*^2^*X* = 51.4%, *Q*^2^ = 26.5%); (d) PLS-DA score plot of MS, NS, and WKN on postdose day 14, showing the degree of separation of the MS (black squares), NS (red dots), and WKN (blue diamonds) (*R*^2^*X* = 23.8%, *R*^2^*Y* = 81.0%, and *Q*^2^ = 38.7%); (e) score plot of the metabolic changing process from PLS-DA model in MS, showing the changing process from predose day −1 (black squares), predose day 7 (red dots), postdose day 7 (shown as day 14, blue diamonds), and postdose day 14 (shown as day 21, green stars)  (*R*^2^*X* = 25.6%, *R*^2^*Y* = 48.2%, and *Q*^2^ = 23.3%); (f) score plot of the metabolic changing process from PLS-DA model in WKN, showing the changing process from predose day −1 (black squares), predose day 7 (red dots), postdose day 7 (shown as day 14, blue diamonds), and postdose day 14 (shown as day 21, green stars) (*R*^2^*X* = 21.5%, *R*^2^*Y* = 55.9%, and *Q*^2^ = 29.7%).

As a supervised method of multivariate statistical analysis to maximize the differences between groups, PLS-DA models were established to assess the metabolic differences between groups with satisfactory discrimination [28]. In the score plot of the PLS-DA model (Figure 2(a)) after model establishment, the MS and NS groups were discriminated obviously with *R*^2^*X* = 51.1%, *R*^2^*Y* = 74.9%, and *Q*^2^ = 51.7%. The parameters of *R*^2^*Y* and *Q*^2^ are used to evaluate the robustness and predictive ability of the PLS-DA model, respectively, and values of more than 0.5 were considered to represent significant differences. Permutation tests are applied to identify an overfitting model, providing cross-validated and self-prediction values, and allowing comparison between the unpermuted results and corresponding permuted results. Based on a 200-iteration permutation test, the validation plot (Figure 2(b)) clarified that the original PLS-DA model was significant and not overfitting as both permuted *R*^2^ and *Q*^2^ values were significantly lower than the corresponding original ones. The significant urinary metabolic variation between the MS and NS groups suggested the FD model was successfully established on predose day 7.

After drug treatment, a PCA model was first constructed to evaluate the separation between the MS, NS, and WKN groups, and a score plot was obtained with the first two PCs performing separately as 51.4% and 26.5% variance (Figure 2(c); *R*^2^*X* = 51.4%, *Q*^2^ = 26.5%). The PCA model showed a good trend of separation between the three groups, but there were also partial overlaps between them. Therefore, PLS-DA was performed to intensify the separation with its distinguished discriminating ability. The PLS-DA score plot of urine samples from the MS, NS, and WKN groups on postdose day 14 showed a clear separation between the three groups with *R*^2^*X* = 23.8%, *R*^2^*Y* = 81.0%, and *Q*^2^ = 38.7% (Figure 2(d)).

A score plot of the metabolic changing process from the PLS-DA model in the MS group, including the metabolic information of predose days −1 and 7 and postdose days 7 and 14, presented the changing process from normal stage, model establishment to intragastric administration of the MS group, shown in Figure 2(e). Meanwhile, the changing process of the WKN group with the same timing points in Figure 2(e) was performed by the PLS-DA score plot of the urine metabolic information in Figure 2(f).

**Figure 3:**
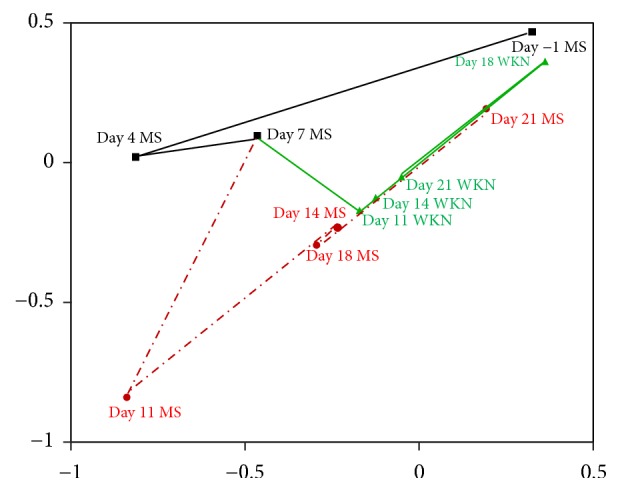
Metabolic trajectory analysis of urine samples from model group (MS) and Weikangning-treated group (WKN) based on PCA model, showing with timing points of predose days −1, 4, and 7 (presented as days −1, 4, and 7) and postdose days 4, 7, 11, and 14 (presented as days 11, 14, 18, and 21), in predose stage there were 20 rats in MS group (black squares), whereas in postdose stage there were 9 rats, respectively, in MS (red dots) and WKN (green triangles) group.

The metabonomic spectra of urine samples from different groups differed from each other, and the occurrence of trajectory bias depending on different intervention methods indicated the differences of changing process. To explore the global changes between the MS and WKN groups, a metabolic trajectory analysis of urine samples from the two groups, including seven timing points (predose days −1, 4, and 7 and postdose days 4, 7, 11, and 14) based on the unsupervised PCA model, is shown in Figure 3. Given that the WKN group was separated from the MS group after the 7-day model establishment at random, the MS and WKN groups were considered as one MS group in the predose stage.

**Figure 4:**
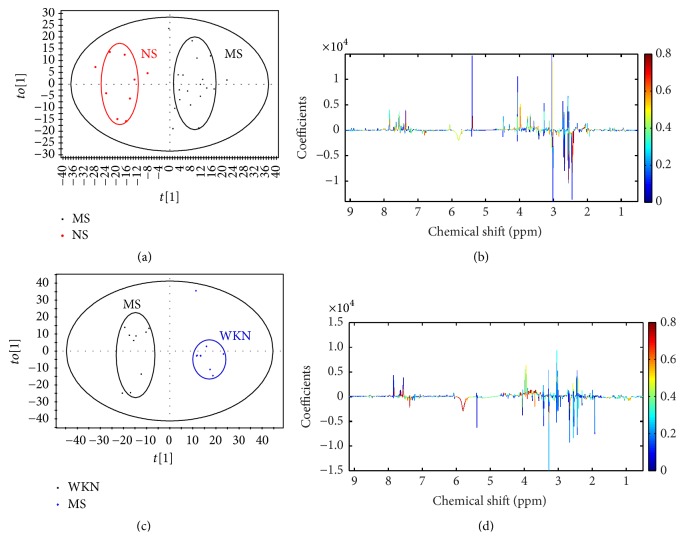
(a) OPLS-DA score plot of model group (MS) and normal control group (NS) on predose day 4, displaying the degree of separation of the model between the MS (black squares) and NS group (red dots) (*R*^2^*X* = 24.2%, *R*^2^*Y* = 83.0%, and *Q*^2^ = 38.9%); (b) OPLS-DA corresponding color-coded correlation coefficient loading plot of key metabolites based on the metabolic information from MS and NS on predose day 4, demonstrating discrimination of key metabolite levels between the two groups; (c) OPLS-DA score plot of MS and Weikangning-treated group (WKN) on postdose day 11, showing the degree of separation between the MS (black squares) and WKN groups (blue diamonds) (*R*^2^*X* = 32.6%, *R*^2^*Y* = 94.2%, and *Q*^2^ = 71.1%); (d) OPLS-DA corresponding color-coded correlation coefficient loading plot of key metabolites based on the metabolic information from MS and WKN groups on postdose day 11, showing differences in levels of key metabolites between the two groups.

According to metabolic trajectory analysis of the urine, metabolic information of the MS group on predose day 4 was further away from normal conditions (predose day −1) than that on predose day 7, and similarly the metabolic information of the WKN group on postdose day 11 was nearest to that of the normal conditions than others. Hence, the predose day 4 and postdose day 11 were selected to be the representative timing points to further study the changed metabolites between MS and NS group and between WKN and MS group separately. Based on the first principal component and the second orthogonal component, OPLS-DA models were built to have an insight into the key metabolites responsible for the separation between the groups. In the OPLS-DA score plots, significant distinctions between the MS and NS group (Figure 4(a)) as well as the WKN and MS group (Figure 4(d)) were identified. The metabolic changes in the MS group compared to those in the NS group, and the WKN group compared to the MS group, were represented in the color-coded coefficient plots (Figures 4(b) and 4(d)). Key metabolites with significant differences (*P* < 0.05) were identified according to the absolute cutoff value of correlation coefficients (|*P*_corr_|) with VIP value and are listed in Tables 1 and 2. With |*P*_corr_| values > 0.60207 (for degree of freedom = 9) and VIP values > 1, the urine samples of the MS group compared to those of the NS group showed upregulation of leucine, lactate, alanine, dimethylamine, phenylacetylglycine, hippurate, allantoin, cytidine, tyrosine, imidazole, and phenylalanine and downregulation of acetate, N-acetylglutamate, succinate, 2-oxoglutarate, citrate, and trimethylamine (Table 1). In another aspect, the urine samples of the WKN group compared to those of the MS group showed upregulation of N-acetylglutamate, glucose, creatine, hippurate, trigonelline, and formate and downregulation of 2-hydroxyvalerate, leucine, alanine, adipate, glycine, cis-aconitate, and phenylalanine under the rules of |*P*_corr_| values > 0.63190 (for degree of freedom = 8) and VIP values > 1 (Table 2). In conclusion, the metabolites of leucine, alanine, and N-acetylglutamate tended to reverse to the normal stage after drug administration.

### 3.5. ^1^H NMR Spectra of Serum Samples

**Figure 5:**
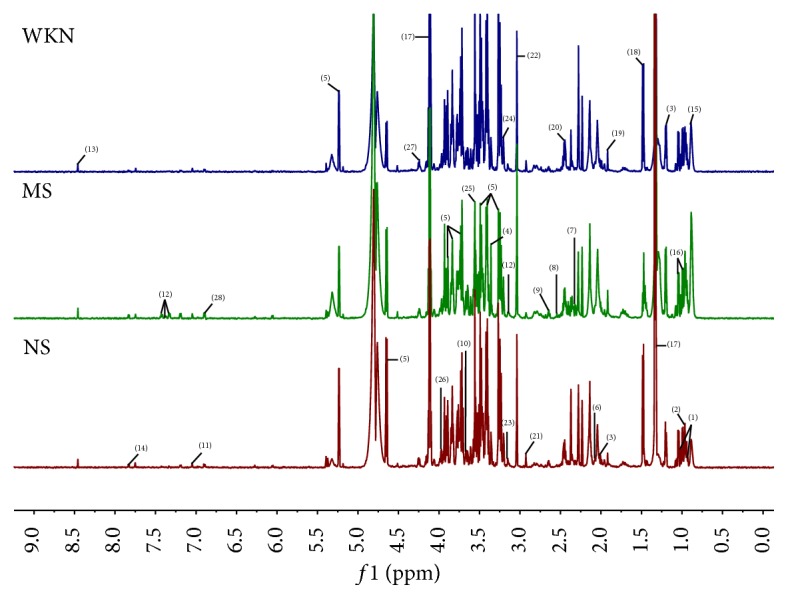
Representative 600 MHz ^1^H NMR spectra of serum samples from model group (MS), normal control group (NS), and Weikangning-treated group (WKN). Distinguished metabolites: (1) isoleucine, (2) leucine, (3) proline, (4) methanol, (5) glucose, (6) glutamate, (7) 3-hydroxybutyrate, (8) citrate, (9) methionine, (10) glycerol, (11) 1-methylhistidine, (12) phenylalanine, (13) formate, (14) tryptophan, (15) 2-hydroxybutyrate, (16) valine, (17) lactate, (18) alanine, (19) acetate, (20) glutamine, (21) dimethylamine, (22) creatinine, (23) histidine, (24) choline, (25) glycine, (26) serine, (27) threonine, and (28) tyrosine.

Representative 600 MHz ^1^H NMR spectra of serum obtained from the MS, NS, and WKN groups are presented in Figure 5. Endogenous metabolites assignments were based on chemical shifts in existing literatures [10, 26]. Several dominant metabolites in serum were identified, including amino acids such as isoleucine, leucine, proline, glutamate, methionine, phenylalanine, and 1-methylhistidine; organic acids such as 3-hydroxybutyrate, and citrate; waste metabolites such as methanol and formate; and glycerol and glucose.

### 3.6. Metabonomic Analysis of Serum Samples

**Figure 6:**
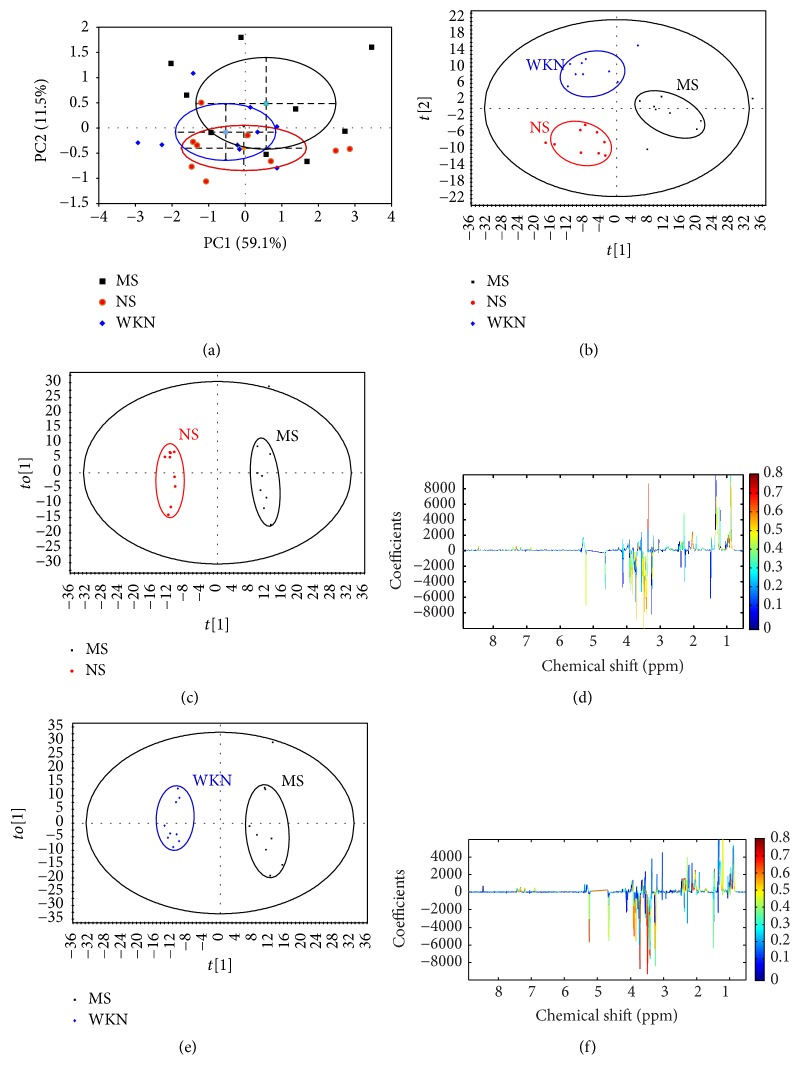
(a) PCA score plot of model group (MS), normal control group (NS), and Weikangning-treated group (WKN) after 14-day drug treatment, performing discrimination between MS (black squares), NS (red dots), and WKN (blue diamonds) (*R*^2^*X* = 59.1%, *Q*^2^ = 11.5%); (b) PLS-DA score plot of MS, NS, and WKN on postdose day 14, showing the degree of separation of the MS (black squares), NS (red dots), and WKN groups (blue diamonds) (*R*^2^*X* = 15.1%, *R*^2^*Y* = 78.0%, and *Q*^2^ = 5.5%); (c) OPLS-DA score plot of model group (MS) and normal control group (NS) on postdose day 14, displaying the degree of separation between the MS (black squares) and NS (red dots) (*R*^2^*X* = 17.7%, *R*^2^*Y* = 99.2%, and *Q*^2^ = 28.9%); (d) OPLS-DA corresponding color-coded correlation coefficient loading plot of key metabolites based on the metabolic information from MS and NS groups on postdose day 14, demonstrating discrimination of key metabolite levels between the two groups; (e) OPLS-DA score plot of MS and WKN-treated (WKN) groups on postdose day 14, showing the degree of separation between the MS (black squares) and WKN groups (blue diamonds) (*R*^2^*X* = 20.1%, *R*^2^*Y* = 97.6%, and *Q*^2^ = 37.2%); (f) OPLS-DA corresponding color-coded correlation coefficient loading plot of key metabolites based on the metabolic information from MS and WKN groups on postdose day 11, showing differences in the levels of key metabolites between the two groups.

According to the signal variability of ^1^H NMR, the PCA model was first established to detect the separation between groups based on serum metabolic information and the score plot of PCA was acquired with the first two PCs showing 59.1% and 11.5% variance separately (Figure 6(a); *R*^2^*X* = 59.1%, *Q*^2^ = 11.5%). To strengthen the ability of separation as the partial overlaps in the PCA score plot between the three groups, a PLS-DA model was constructed to evaluate the relative metabolic differences on postdose day 14 and exhibited good ability to discriminate between the MS, NS, and WKN groups, shown in the relative score plot in Figure 6(b).

To detect the key metabolites playing important roles in the separation between groups from serum samples after administration, OPLS-DA models were established between the MS, NS, and WKN groups, using the first principal and second orthogonal components. In the OPLS-DA score plots, significant distinctions within the three groups (Figures 6(c) and 6(e)) through the paired-comparisons were identified. The corresponding metabolic changes in the MS and WKN groups by comparison with the NS and MS groups, respectively, were performed using color-coded coefficient plots (Figures 6(d) and 6(f)). Key metabolites exhibiting significant changes (*P* < 0.05) were identified in accordance with the absolute cutoff value of correlation coefficients (|*P*_corr_|) and VIP value, presented in Tables 3 and 4. With |*P*_corr_| values > 0.63190 (for degree of freedom = 8) and VIP values > 1, the serum samples of the MS group compared to those of the NS group manifested upregulation of isoleucine, leucine, proline, and methanol and downregulation of glucose (Table 3). According to the same rules of |*P*_corr_| values and VIP values, the serum samples of the WKN group compared to those of the MS group exhibited upregulation of glucose and downregulation of proline, glutamate, 3-hydroxybutyrate, citrate, methionine, glycerol, 1-methylhistidine, phenylalanine, and formate (Table 4). After drug administration, the metabolites of glucose and proline had the tendency to reverse to the normal stage.

### 3.7. Metabolic Pathway and Function Analysis

**Figure 7:**
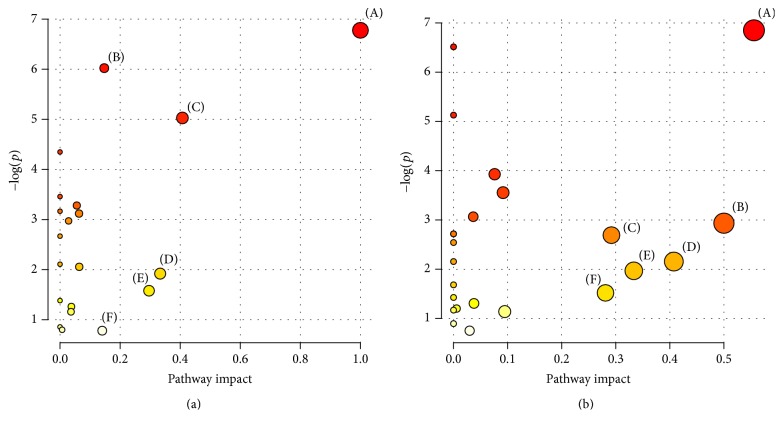
(a) Summary of pathway analysis with MetPA in model establishment: (A) phenylalanine, tyrosine, and tryptophan biosynthesis, (B) citrate cycle (TCA cycle or Krebs cycle), (C) phenylalanine metabolism, (D) valine, leucine and isoleucine biosynthesis, (E) glyoxylate and dicarboxylate metabolism, and (F) tyrosine metabolism; (b) summary of pathway analysis with MetPA in drug intervention: (A) glyoxylate and dicarboxylate metabolism, (B) phenylalanine, tyrosine and tryptophan biosynthesis, (C) glycine, serine, and threonine metabolism, (D) phenylalanine metabolism, (E) valine, leucine, and isoleucine biosynthesis, and (F) glycerolipid metabolism.

Combining the results of identified key metabolites in urine and serum samples, the metabolic pathway analyses were performed using MetPA (Metabolomics Pathway Analysis) to uncover the most relevant pathways involved during the FD model establishment and following drug intervention. The potential target pathways were identified with the impact value calculated from the pathway topology analysis being above 0.1. According to these parameters, there were six potential target pathways (phenylalanine, tyrosine and tryptophan biosynthesis, citrate cycle (TCA cycle or Krebs cycle), phenylalanine metabolism, valine, leucine and isoleucine biosynthesis, glyoxylate and dicarboxylate metabolism, and tyrosine metabolism) found to be active in the period of model establishment (Figure 7(a); Table S1). However, six potential target pathways (glyoxylate and dicarboxylate metabolism; phenylalanine, tyrosine, and tryptophan biosynthesis; glycine, serine, and threonine metabolism; phenylalanine metabolism; valine, leucine, and isoleucine biosynthesis; and glycerolipid metabolism) were identified during the period of drug treatment (Figure 7(b); Table S2). Four of these pathways overlapped with those found in the modeling process, indicating that the mechanisms of action of WKN involved more than one target.

## 4. Discussion

Up to 42% of patients with FD consult a physician, whereas many people who suffer from the disease do not seek medical advice, and this condition negatively impacts on work productivity due to poor appetite and malnourishment [2, 29]. Also, there is a heavy economic burden on patients and society due to the limited efficacy of the majority of conventional medical therapies available. Additionally, the negative psychosocial impacts of the condition greatly lower the quality of life of FD patients, as demonstrated by the high comorbidity with anxiety and depression (33–77%). In the meantime, recent pathophysiological studies have increased new insights into the pathogenesis of FD [30].

**Figure 8:**
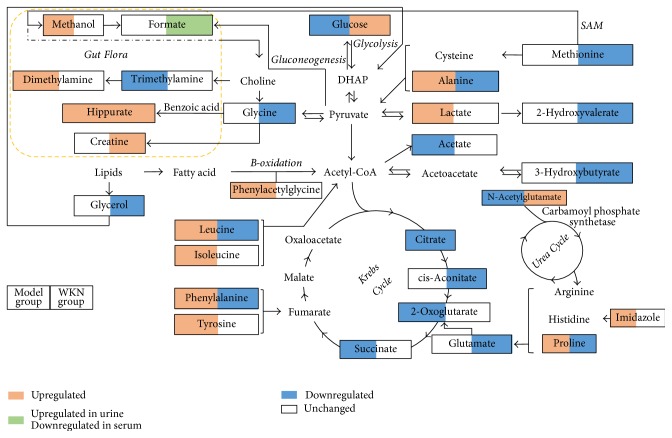
Schematic diagram of the disturbed metabolic network detected by ^1^H NMR analysis showing the interrelationship of the identified metabolic pathways. The pink squares represent significant upregulation of metabolites in model group compared to in the normal control group (left section) or in WKN-treated group (WKN group) compared to in the model group (right section), whereas the blue squares indicate downregulation. Green squares demonstrate upregulation in urine with downregulation in serum; the white squares represent a lack of significant change. DHAP: dihydroxyacetone phosphate; SAM: S-adenosyl methionine.

In this present work, we adopted stressed rat model via tail damping to established FD model which was verified in our previous research with convincing results [13]. After 7-day model establishment, significant urinary metabolic variation between MS and NS group was exhibited through the results of PLS-DA model (*R*^2^*X* = 51.1%, *R*^2^*Y* = 74.9%, and *Q*^2^ = 51.7%) and the corresponding permutation test, proving the FD model was achieved in accordance with the behavior observation and significantly reduced daily food and water intake of the MS group. After drug treatment, PCA models were constructed to evaluate the separation between the MS, NS, and WKN-treated groups. With the results of the first two PCs performing 51.4% and 26.5% variance and 59.1% and 11.5% variance based on urine and serum metabolic information, respectively, PCA models demonstrated a good trend of separation between the three groups. Then, for the identification of key metabolites responsible for the differentiation within groups, OPLS-DA models were established between the three groups by paired-comparisons with high reliability. Under the guidance of urinary metabolic trajectory, 17 potential biomarkers in the development of the FD model were detected in the contrast between the MS and NS groups, where 13 key metabolites based on the urine samples of the WKN and MS groups were determined in drug treatment, and five of them (leucine, alanine, phenylalanine, N-acetylglutamate, and hippurate) overlapped with the discovery in the FD modeling. However, 5 key metabolites were identified in the comparison of differences in serum metabolic information between the MS and NS groups, and 10 potential biomarkers were selected to represent the metabolic changes in the MS group treated with WKN and two of them (proline and glucose) overlapped with the former. With the help of MetPA and identified key metabolites of the two biofluids, six potential target pathways were found, respectively, in the processes of FD model establishment and drug intervention to reveal the disturbed metabolic network (Figure 8).

### 4.1. Metabolites Related to Energy Metabolism

Due to the overlap of four metabolic pathways, there were eight most relevant pathways involved in metabolic profiling. According to the KEGG metabolism network, two of them (citrate cycle and glyoxylate and dicarboxylate metabolism) belong to carbohydrate metabolism, one (glycerolipid metabolism) belongs to lipid metabolism, and five of them (phenylalanine metabolism, phenylalanine, tyrosine, and tryptophan biosynthesis, valine, leucine, and isoleucine biosynthesis, tyrosine metabolism, and glycine, serine, and threonine metabolism) are in the range of amino acid metabolism. Given that carbohydrate, lipids, and proteins are the three basic energy substances, their metabolism is closely associated with energy metabolism and the citrate cycle (TCA cycle or Krebs cycle), and their shared metabolic pathways play an important and crucial role in the energy metabolic network [31]. In FD modeling, decreased levels of glucose, acetate, and citrate cycle products (citrate, 2-oxoglutarate, and succinate) and increased levels of amino acids (leucine, isoleucine, tyrosine, phenylalanine, alanine, and proline) indicate a high demand for and rapid utilization of metabolites to fulfill energy producing pathways in accordance with the reduced food intake of the MS group [32, 33]. Disturbed energy metabolism is also reflected in the high levels of lactate as a result of accelerated glycolysis to feed the energy demand of increased exercise in the FD model rats to escape from tail stimulation [34]. The results differ slightly from an observation of higher levels of proline and lower levels of *β*-glucose, lactate, and leucine/isoleucine in FD patients compared with those of healthy control presented in a recent clinical study based on NMR metabolic profiling [35]. Considering the chronic and relapsing natural course of FD, decreased lactate and leucine/isoleucine could be the result of metabolic consumption in the long term conditions of starvation and malnutrition [33, 36]. However, with the high degree of consistency in the variation tendency of metabolites, our results give an insight into the disturbance of the energy metabolic system in the primary stage of FD. Similarly, our previous work demonstrated abnormal glycometabolism and lipid metabolism in upper gastrointestinal (GI) tract tissues of FD model rats due to insufficient energy supply [13]. As a consequence of the treatment of WKN, our study detected a high level of glucose and low levels of Krebs cycle products (citrate and cis-aconitate), amino acids (alanine, methionine, glycine, leucine, phenylalanine, glutamate, and proline), 2-hydroxyvalerate, 3-hydroxybutyrate, and glycerol, indicating increased gluconeogenesis and decreased degradation of proteins and lipids with reduced glycolysis. Additionally, the elimination of amino acid nitrogen is mainly through the urea cycle, and N-acetylglutamate, as the essential activator of carbamoyl phosphate synthetase (which is the first enzyme in urea production), mediates the urea biosynthesis [37]. The alteration of N-acetylglutamate level is in response to protein ingestion, indicating a changed nutritional status in model rats. These outcomes reveal that energy metabolism is a therapeutic target of WKN and provide evidence of the improvement in the appetite of FD model rats treated by WKN.

### 4.2. Amino Acids and Stress-Related Metabolites

Despite serving as energy sources, amino acids also have a variety of curial biological functions in living systems [38]. Stress, described as the general response of the body to any noxious stimulus, stimulates the hypothalamic-pituitary-adrenal (HPA) axis and sympathomedullary axis as the two dominant stress response pathways [39, 40]. The sympathomedullary pathway is responsible for the production of the fight of flight response mainly in short term stress, and the activation of this axis results in the release of catecholamines, including dopamine, norepinephrine, and epinephrine, involved in a variety of alterations in neurophysiology such as the regulation of mood, anxiety, and even appetitive motivation [41, 42]. Phenylalanine and its metabolite tyrosine, as catecholamine precursors, participate in dopamine synthesis and are associated with excessive stimulation of the sympathetic nervous system [38]. The changes of phenylalanine and tyrosine in stressed rats might be a response to the tail stimulation via the sympathomedullary axis to prevent the overconsumption of dopamine, which may lead to adverse effects on mental functions. Neurotransmitters also play significant roles in the transfer of information in the central and peripheral nervous systems. Glutamate (as excitatory neurotransmitter), glycine, and alanine (as inhibitory neurotransmitters) are central players in the maintenance of normal brain function and the improvement of several neurological disorders [43–45]. The branched-chain amino acids (BCAAs) including valine, leucine, and isoleucine in similar metabolic processes have been found to be involved in stress, energy, and muscle metabolism and also have neurophysiological therapeutic effects related to the competitive relationship with aromatic amino acids (ArAAs) responsible for the release of several neurotransmitters, notably serotonin (from tryptophan), and catecholamines (form phenylalanine and tyrosine) [46]. The upregulation of BCAAs contributes to a reduction in the uptake of tryptophan and the synthesis of serotonin and may play a role in delaying central fatigue [47]. In addition, it has been reported that BCAAs can enhance glutathione S-transferase (GST) and catalase activities to exert antioxidative effects against tissue oxidative stress in intestinal epithelial cells [48]. Besides, the uric acid (UA) cycle is one of the defensive antioxidant subsystems, and UA is mainly degraded to allantoin by urate oxidase in most mammals [49]. High levels of allantoin may be considered as a response to increased oxidative stress, and gut flora takes part in the metabolic process of allantoin [50, 51]. Increased creatine has been discovered in oxidative stress but with therapeutic effects, and the degradation of creatine involves intestinal bacteria as well [52, 53]. However, elevated creatine levels could be a reflection of an increased glomerular filtration rate caused by the WKN treatment. The changes of stress-associated metabolites might indicate an overall stress condition in the model rats and show the potential antioxidant effects of WKN. Also, in our previous work, the status of oxidative stress in FD rats and the protective effects of WKN were revealed by the alterations of superoxide dismutase 2 (SOD2) and glutathione S-transferase pi2 (GSTP2) with the use of proteomic method [13].

### 4.3. Metabolites of the Gut Microbiota

It is well known that stress can lead to a variety of alterations in the GI tract in relation to but not limited to intestinal motility, visceral hypersensitivity, mucosal transport, and gut barrier function and can also affect gut flora through physical, immune, and neurochemical mechanisms [54]. Meanwhile, gut microbiota can reflect various physiological functions from energy metabolism to mental status and at the same time have influences on health and disease conditions of the host, including neurodevelopment and central nervous system (CNS) performance, via a series of metabolic and immune regulatory axes [55]. Thus, the concept of a microbiome-brain-gut axis has emerged to describe the interactions among the three and has begun to attract more and more attention for its importance and complexity. Methylamines, including trimethylamine (TMA), trimethylamine N-oxide (TMAO), and dimethylamine (DMA) yielded from TMA through demethylation, are products of dietary choline metabolized by intestinal microbiota [56]. Alterations in DMA and TMA are considered to be related to several metabolic disorders, such as nonalcoholic fatty liver disease, obesity, and diabetes [57]. Hippurate, a known glycine conjugate of benzoic acid, is related to the metabolic processes of phenyl derivatives involved in the diet and degraded by gut microbiota and can be used as a urinary biomarker of obesity and hypertension in humans [58]. Similarly, phenylacetylglycine (PAG), formed by the conjugation of phenylacetyl-CoA with glycine and regarded as a surrogate biomarker for phospholipidosis [59], correlates with the production of phenylacetate, which is influenced by the *β*-oxidation of fatty acids, the metabolic formation of phenylalanine, and gut flora [60]. Moreover, it is worth noting that increased methanol is detected in the serum of FD model rats. Metabolic methanol may occur physiologically as a result of fermentation by gut bacteria, and the maintenance of low methanol levels facilitates physiological and metabolic clearance mechanisms [61]. Formaldehyde, the short-lived oxidized product of methanol, is found to be elevated in the blood of neurological patients as a putative causative agent [62]. Hence, the upregulation of methanol as well as DMA, hippuate, and PAG may result from the interactions among diet, gut microbial activity, and psychological distress. Furthermore, because formate can be converted from methanol through formaldehyde primarily by alcohol dehydrogenases and can also be produced by gut microbiota [63], the increased levels of formate in urine and decreased levels in serum in the WKN group may reflect the intervention effects on gut microbiota and metabolic clearance mechanisms that are involved in WKN's effects in FD.

The disturbances of energy metabolism, stress-related metabolism, and gut microbiota in stressed FD model rats mentioned above are generally consistent with the previous findings of metabolic profiling in human subjects with high anxiety trait [64]. Our observations also suggest the potential beneficial effects of WKN in FD for improving the conditions of diet, intestinal microbiota, and mental health which may relate to the regulation of the microbiome-gut-brain axis. In addition, the elevated level of trigonelline in urine samples, the major alkaloid in* Rhizome Pinelliae Preparata,* one of the herbs in WKN, may be the result of drug administration [65]. Several researchers have discussed the effect of trigonelline on reducing glucose concentrations and how it may contribute to the beneficial impact of coffee in Type-2 Diabetes [66].

However, according to the metabolic trajectory analysis of urine, several remaining issues need to be explored through further study. Firstly, with a limited duration of stress stimulation and the relapsing but remitting natural course of FGIDs, there was a trend to be normalized toward the normal condition in the MS group, and a similar observation in the urinary metabolic signatures of humans with high anxiety strait has been reported in the literature [64]. This condition may influence the best treatment duration and may require a shorter intervention time, such as seven to ten days, in stress-related experimental studies of FGIDs, considering that the MS group was nearer to the normal condition than the WKN group on postdose day 14. Besides, the MS group is greatly different from the normal condition in the second time on postdose day 4, which implied gastric intubation might be a stressor itself to laboratorial rats with alterations of endogenous metabolites detected by metabonomic analysis sensitively. On the one hand, with the comparison between the MS and WKN groups on postdose day 4 in the metabolic trajectory analysis of urine, WKN shows potential advantages in decreasing mental stress and improving the metabolic response to stress. However, alternative methods of drug administration for the replacement of gastric intubation need to be explored in future studies.

## 5. Conclusions

In the present work, the ^1^H NMR-based metabonomic method in conjunction with multivariate data analysis was performed to explore the metabolic phenotype of FD in stressed rats and evaluate the intervention effect of WKN as well as the underlying pharmacodynamics involved. The current observations in both urine and serum samples indicate that the disorder of energy metabolism, stress-related metabolism, and gut microbiota was caused by stress stimulation in FD model rats, and this condition was restored to normal after WKN administration. This study may provide new insights into the relationship between FGIDs and mental stress based on metabolic profiling and assist in the future clinical evaluation of FD and uncovering of the mechanisms of action involved in Chinese medicines.

## Supplementary Material

Figure S1 shows the identified results of baicalin and berberine hydrochloride based on HPLC; Figure S2 represents the comparisons in daily food and water intake and body weights between the model and normal control groups after 7-day modeling; Figure S3 reveals the comparisons in daily food and water intake and body weights between the Weikangning-treated, model and normal control groups after 14-day drug administration; Figure S4 shows Stomach histopathology for model assessment; Table S1 contains the results from Pathway Analysis with MetPA in model establishment; Table S2 exhibits the results from Pathway Analysis with MetPA in drug treatment.

## Figures and Tables

**Table 1:** Quantitative comparison of metabolites identified in urine of model group (MS) and normal control group (NS).

Metabolites	Chemical shift	Integral in MS group^a^ (mean ± S.D.) × 10^−2^	Integral in NS group^a^ (mean ± S.D.) × 10^−2^	*P*_corr_^b^	VIP	*P* ^c^
				(|*P*_corr_ | > 0.602)		(*P* < 0.05)
Leucine	0.95 (t)	10.112 ± 1.155	8.732 ± 1.004	0.659 (↑)	1.779	0.004
Lactate	1.335 (d)	8.332 ± 0.997	7.284 ± 0.951	0.648 (↑)	1.845	0.011
Alanine	1.485 (d)	6.328 ± 0.539	5.582 ± 0.614	0.633 (↑)	2.135	0.002
Acetate	1.93 (s)	7.851 ± 2.285^d^	14.826 ± 27.119^d^	−0.634 (↓)	1.735	0.031^d^
N-Acetylglutamate	2.225 (t)	10.518 ± 0.978	12.481 ± 1.975	−0.650 (↓)	1.981	0.013^c′^
Succinate	2.41 (s)	16.494 ± 5.225	27.520 ± 12.593	−0.704 (↓)	1.996	0.023^c′^
2-Oxoglutarate	**2.46 (t)**, 3.015 (t)	48.701 ± 16.029	72.679 ± 25.975	−0.698 (↓)	1.966	0.005
Citrate	2.565 (AB)	21.411 ± 40.034^d^	73.047 ± 45.443^d^	−0.643 (↓)	2.166	0.004^d^
Dimethylamine	2.725 (s)	64.191 ± 12.555	48.890 ± 9.897	0.656 (↑)	1.951	0.002
Trimethylamine	2.92 (s)	3.671 ± 0.718	4.701 ± 0.948	−0.676 (↓)	1.753	0.003
Phenylacetylglycine	3.68 (s)	34.812 ± 6.582	27.550 ± 3.781	0.694 (↑)	1.794	0.003
Hippurate	**3.965 (t)**, 7.71 (d), 7.83 (d)	17.012 ± 2.050	14.239 ± 1.772	0.697 (↑)	1.897	0.001
Allantoin	5.39 (s)	117.921 ± 32.112^d^	88.265 ± 30.123	0.707 (↑)	1.623	0.035^d^
Cytidine	6.07 (d)	11.707 ± 2.572	7.857 ± 1.949	0.666 (↑)	1.968	0.000
Tyrosine	7.28 (s)	7.227 ± 1.931^d^	5.411 ± 1.349	0.661 (↑)	1.618	0.009^d^
Imidazole	7.365 (t)	25.161 ± 7.959	15.153 ± 3.917	0.715 (↑)	1.888	0.001
Phenylalanine	7.425 (t)	9.045 ± 2.891	5.604 ± 1.378	0.694 (↑)	1.782	0.001

^a^The relative integrals of metabolites were determined by 1D ^1^H NMR analysis of urine. ^b^The values of the correlation number extracted from the correlation plots of OPLS-DA models of MS versus NS. ^c^The *P* values were obtained from Independent-Samples *t*-test or *t*′-test (shown with c′). ^d^The *P* values were calculated by Mann–Whitney *U* test of nonparametric tests and the related integrals were presented in median ± IQR The arrows (↑/↓) denote higher/lower amounts relative to NS group.

**Table 2:** Quantitative comparison of metabolites identified in urine of Weikangning-treated group (WKN) and model group (MS).

Metabolites	Chemical shift	Integral in WKN group^a^ (mean ± S.D.) × 10^−2^	Integral in MS group^a^ (mean ± S.D.) × 10^−2^	*P*_corr_^b^ (|*P*_corr_ | > 0.632)	VIP	*P* ^c^ (*P* < 0.05)
2-Hydroxyvalerate	0.91 (t)	4.087 ± 1.180^d^	5.443 ± 1.093	−0.641 (↓)	1.762	0.041^d^
Leucine	0.95 (dd)	5.711 ± 0.857	8.219 ± 1.782^d^	−0.678 (↓)	1.884	0.001^d^
Alanine	1.48 (d)	3.558 ± 0.345	4.877 ± 0.981	−0.704 (↓)	1.744	0.003^c′^
Adipate	1.555 (m)	5.492 ± 0.653	6.826 ± 1.032	−0.645 (↓)	1.816	0.003
N-Acetylglutamate	2.22 (t)	22.047 ± 7.360	7.318 ± 0.913	0.826 (↑)	2.267	0.000^c′^
Glucose	3.525 (dd), **3.82 (ddd, m)**	21.412 ± 2.625	15.989 ± 1.042	0.930 (↑)	2.397	0.000^c′^
Glycine	3.57 (s)	9.757 ± 0.994	12.134 ± 2.001	−0.727 (↓)	1.625	0.005^c′^
Creatine	3.925 (t)	13.164 ± 1.173	11.213 ± 1.026	0.801 (↑)	1.878	0.001
Hippurate	**3.965 (d)**, 7.555 (t), 7.63 (t), 7.83 (t)	21.440 ± 4.307	15.534 ± 2.023	0.708 (↑)	2.027	0.002^c′^
Trigonelline	4.435 (s)	4.575 ± 1.232	3.358 ± 0.785	0.657 (↑)	1.635	0.017
cis-Aconitate	5.69 (s)	2.330 ± 0.932	4.625 ± 2.431	−0.661 (↓)	1.498	0.017^c′^
Phenylalanine	7.42 (t)	4.416 ± 1.583	6.770 ± 2.040	−0.688 (↓)	1.856	0.010
Formate	8.46 (s)	0.970 ± 0.292	0.387 ± 0.102	0.803 (↑)	2.289	0.000^c′^

^a^The relative integrals of metabolites were determined by 1D ^1^H NMR analysis of urine. ^b^The values of the correlation number extracted from the correlation plots of OPLS-DA models of WKN versus MS groups. ^c^The *P* values were obtained from Independent-Samples *t*-test or *t*′-test (shown with c′). ^d^The *P* values were calculated by Mann–Whitney *U* test of nonparametric tests and the related integrals were presented in median ± IQR. The arrows (↑/↓) denote higher/lower amounts relative to MS group.

**Table 3:** Quantitative comparison of metabolites identified in serum of model group (MS) and normal control group (NS).

Metabolites	Chemical shift	Integral in MS group^a^ (mean ± S.D.) × 10^−2^	Integral in NS group^a^ (mean ± S.D.) × 10^−2^	*P*_corr_^b^ (|*P*_corr_| > 0.632)	VIP	*P* ^c^ (*P* < 0.05)
Isoleucine	0.94 (t)	0.146 ± 0.037	0.116 ± 0.020	0.702 (↑)	2.350	0.035
Leucine	0.96 (dd)	0.220 ± 0.043^d^	0.193 ± 0.019^d^	0.693 (↑)	2.368	0.010^d^
Proline	2.07 (m)	0.085 ± 0.014	0.067 ± 0.006	0.661 (↑)	2.227	0.002^c′^
Methanol	3.36 (s)	0.385 ± 0.165	0.195 ± 0.032	0.769 (↑)	2.674	0.005
Glucose	3.705 (t)	0.314 ± 0.114	0.425 ± 0.090	−0.642 (↓)	2.237	0.026

^a^The relative integrals of metabolites were determined by 1D ^1^H NMR analysis of urine. ^b^The values of the correlation number extracted from the correlation plots of OPLS-DA models of MS versus NS groups. ^c^The *P* values were obtained from Independent-Samples *t*-test or *t*′-test (shown with c′). ^d^The *P* values were calculated by Mann–Whitney *U* test of nonparametric tests and the related integrals were presented in median ± IQR. The arrows (↑/↓) denote higher/lower amounts relative to NS group.

**Table 4:** Quantitative comparison of metabolites identified in serum of Weikangning-treated group (WKN) and model group (MS).

Metabolites	Chemical shift	Integral in WKN group^a^ (mean ± S.D.) × 10^−2^	Integral in MS group^a^ (mean ± S.D.) × 10^−2^	*P*_corr_^b^ (|*P*_corr_ | > 0.632)	VIP	*P* ^c^ (*P* < 0.05)
Proline	2.04 (m)	0.300 ± 0.040	0.357 ± 0.069^d^	0.660 (↓)	2.535	0.023^d^
Glutamate	2.105 (m)	0.040 ± 0.005	0.052 ± 0.011^d^	0.656 (↓)	2.175	0.001^d^
3-Hydroxybutyrate	2.355 (m)	0.080 ± 0.013	0.112 ± 0.028	0.730 (↓)	2.320	0.008^c′^
Citrate	2.555 (AB)	0.008 ± 0.002	0.012 ± 0.002	0.707 (↓)	2.282	0.001
Methionine	2.645 (t)	0.034 ± 0.006	0.042 ± 0.010	0.739 (↓)	2.430	0.045
Glucose	**3.44 (t)**, 3.5 (t), 3.725 (dd), 3.9 (dd)	0.120 ± 0.013	0.096 ± 0.022^d^	−0.736 (↑)	2.430	0.010^d^
Glycerol	3.67 (ABX)	0.134 ± 0.014	0.165 ± 0.047^d^	0.697 (↓)	2.160	0.041^d^
1-Methylhistidine	7.04 (s)	0.000 ± 0.001	0.003 ± 0.002	0.639 (↓)	2.242	0.007
Phenylalanine	7.32 (m), **7.375 (m)**	0.000 ± 0.002	0.004 ± 0.007^d^	0.668 (↓)	2.374	0.002^d^
Formate	8.46 (s)	0.038 ± 0.006	0.058 ± 0.023^d^	0.714 (↓)	2.591	0.001^d^

^a^The relative integrals of metabolites were determined by 1D ^1^H NMR analysis of urine. ^b^The values of the correlation number extracted from the correlation plots of OPLS-DA models of WKN versus MS groups. ^c^The *P* values were obtained from Independent-Samples *t*-test or *t*′-test (shown with c′). ^d^The *P* values were calculated by Mann–Whitney *U* test of nonparametric tests and the related integrals were presented in median ± IQR. The arrows (↑/↓) denote higher/lower amounts relative to MS group.
