# Supplementary material for: 1H NMR-Based Metabonomic Study of Functional Dyspepsia in Stressed Rats Treated with Chinese Medicine Weikangning
Source: Evid Based Complement Alternat Med. 2017 Sep 28;2017:4039425. doi: 10.1155/2017/4039425 (PMC5637829; doi:10.1155/2017/4039425)
Supplement: Supplementary file 1 — Figure S1 shows the identified results of baicalin and berberine hydrochloride based on HPLC; Figure S2 represents the comparisons in daily food and water intake and body weights between the model and normal control groups after 7-day modeling; Figure S3 reveals the comparisons in daily food and water intake and body weights between the Weikangning-treated, model and normal control groups after 14-day drug administration; Figure S4 shows Stomach histopathology for model assessment; Table S1 contains the results from Pathway Analysis with MetPA in model establishment; Table S2 exhibits the results from Pathway Analysis with MetPA in drug treatment. [file 4039425.f1.docx]

**Electronic Supplementary Information**

**Figures**


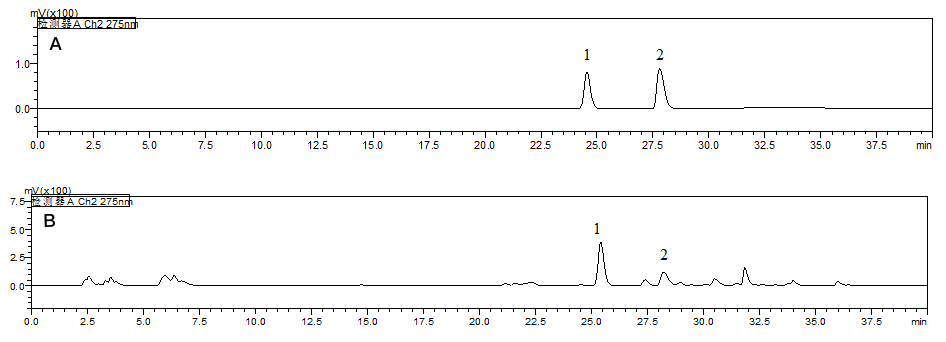


Figure S1. The identified results of *baicalin (1)* and *berberine hydrochloride (2)*. (A) HPLC chromatogram of standard samples; (B) HPLC chromatogram of Weikangning.


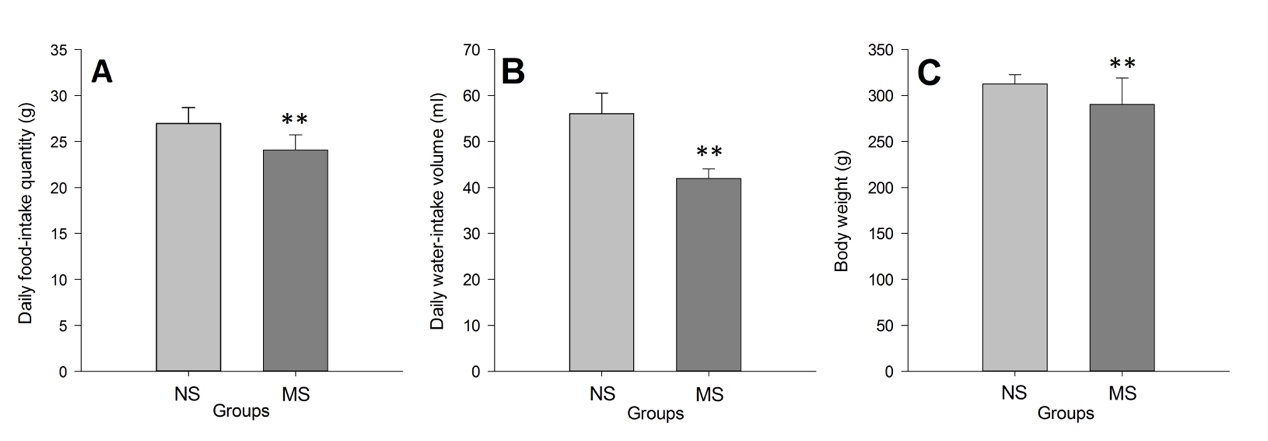


Figure S2. Daily food and water intake and body weights of rats from model group (MS, n=20) and normal control group (NS, n=10) were expressed as mean ± S.D.; (A) Daily food-intake quantity (g), (B) Daily water-intake volume (ml), (C) Body weight (g); compared with NS group **p<0.01.


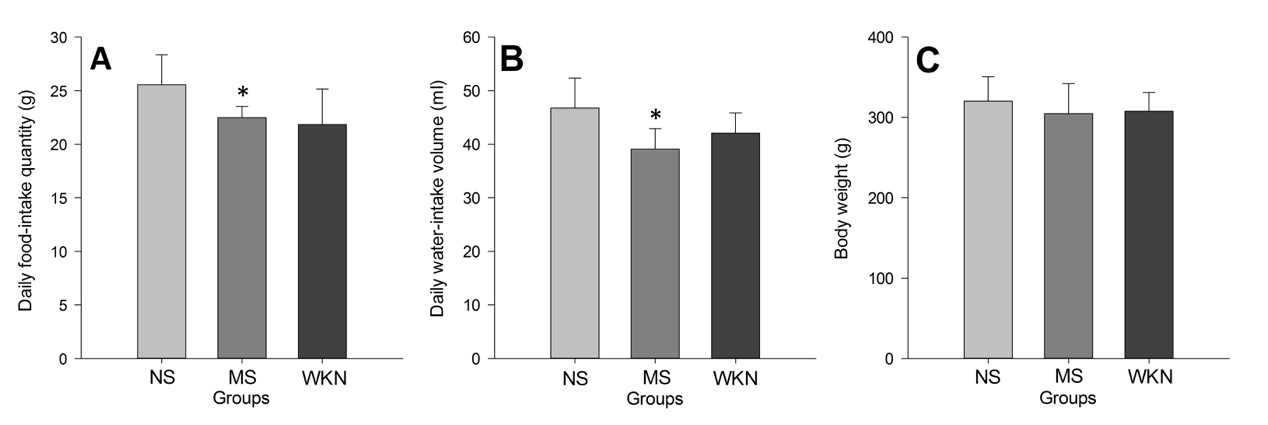


Figure S3. Daily food and water intake and body weights of rats from model group (MS, n=9), normal control group (NS, n=9) and Weikangning-treated group (WKN) and were expressed as mean ± S.D.; (A) Daily food-intake quantity (g), (B) Daily water-intake volume (ml), (C) Body weight (g); compared with NS group *p<0.05.


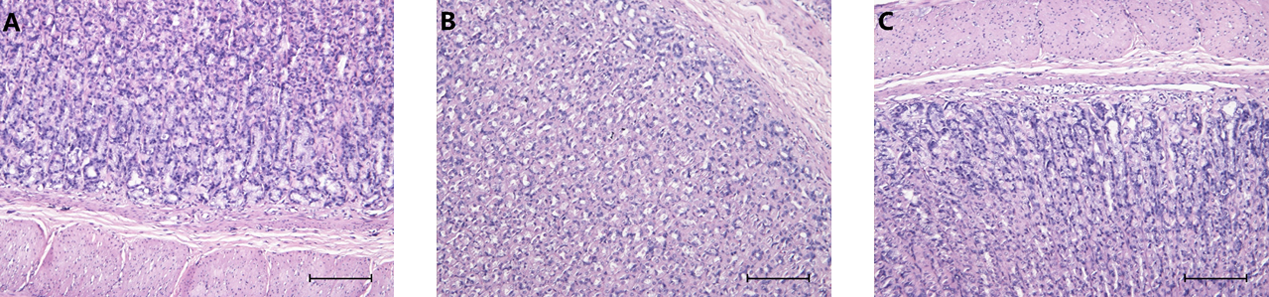


Figure S4. Stomach histopathology of rats from model group (A and B) and normal control group (C). (HE, ×200, bar = 200μm)

**Tables**

Table S1 Results from Pathway Analysis with MetPA in model establishment

|  | Total | Expected | Hits | Raw p | =-LOG(p) | Holm adjust | FDR | Impact |
| --- | --- | --- | --- | --- | --- | --- | --- | --- |
| Phenylalanine, tyrosine and tryptophan biosynthesis | 4 | 0.057061 | 2 | 0.001141 | 6.7759 | 0.092418 | 0.092418 | 1 |
| Citrate cycle (TCA cycle) | 20 | 0.28531 | 3 | 0.002427 | 6.0211 | 0.19417 | 0.098296 | 0.14721 |
| Phenylalanine metabolism | 9 | 0.12839 | 2 | 0.006558 | 5.0271 | 0.51808 | 0.17707 | 0.40741 |
| Aminoacyl-tRNA biosynthesis | 67 | 0.95578 | 4 | 0.012935 | 4.3478 | 1 | 0.26194 | 0 |
| Butanoate metabolism | 20 | 0.28531 | 2 | 0.031505 | 3.4576 | 1 | 0.44787 | 0 |
| Pyruvate metabolism | 22 | 0.31384 | 2 | 0.037657 | 3.2792 | 1 | 0.44787 | 0.05583 |
| Ubiquinone and other terpenoid-quinone biosynthesis | 3 | 0.042796 | 1 | 0.042218 | 3.1649 | 1 | 0.44787 | 0 |
| Alanine, aspartate and glutamate metabolism | 24 | 0.34237 | 2 | 0.044234 | 3.1183 | 1 | 0.44787 | 0.06329 |
| Glycolysis or Gluconeogenesis | 26 | 0.3709 | 2 | 0.05121 | 2.9718 | 1 | 0.46089 | 0.02862 |
| D-Glutamine and D-glutamate metabolism | 5 | 0.071327 | 1 | 0.069417 | 2.6676 | 1 | 0.56228 | 0 |
| Methane metabolism | 9 | 0.12839 | 1 | 0.12163 | 2.1068 | 1 | 0.8644 | 0 |
| Arginine and proline metabolism | 44 | 0.62767 | 2 | 0.12806 | 2.0553 | 1 | 0.8644 | 0.064 |
| Valine, leucine and isoleucine biosynthesis | 11 | 0.15692 | 1 | 0.14668 | 1.9195 | 1 | 0.91392 | 0.33333 |
| Glyoxylate and dicarboxylate metabolism | 16 | 0.22825 | 1 | 0.20637 | 1.5781 | 1 | 1 | 0.2963 |
| Propanoate metabolism | 20 | 0.28531 | 1 | 0.25124 | 1.3813 | 1 | 1 | 0 |
| Starch and sucrose metabolism | 23 | 0.3281 | 1 | 0.28331 | 1.2612 | 1 | 1 | 0.03778 |
| Galactose metabolism | 26 | 0.3709 | 1 | 0.31406 | 1.1582 | 1 | 1 | 0.03644 |
| Valine, leucine and isoleucine degradation | 38 | 0.54208 | 1 | 0.42499 | 0.85568 | 1 | 1 | 0 |
|  | Total | Expected | Hits | Raw p | =-LOG(p) | Holm adjust | FDR | Impact |
| Pyrimidine metabolism | 41 | 0.58488 | 1 | 0.44994 | 0.79865 | 1 | 1 | 0.00709 |
| Tyrosine metabolism | 42 | 0.59914 | 1 | 0.45802 | 0.78084 | 1 | 1 | 0.14045 |

Table S2 Results from Pathway Analysis with MetPA in drug treatment

|  | Total | Expected | Hits | Raw p | =-LOG(p) | Holm adjust | FDR | Impact |
| --- | --- | --- | --- | --- | --- | --- | --- | --- |
| Glyoxylate and dicarboxylate metabolism | 16 | 0.21683 | 3 | 0.001059 | 6.8508 | 0.085744 | 0.059986 | 0.55556 |
| Aminoacyl-tRNA biosynthesis | 67 | 0.90799 | 5 | 0.001481 | 6.5149 | 0.11849 | 0.059986 | 0 |
| Methane metabolism | 9 | 0.12197 | 2 | 0.005922 | 5.1291 | 0.46784 | 0.15989 | 0 |
| Arginine and proline metabolism | 44 | 0.59629 | 3 | 0.019674 | 3.9285 | 1 | 0.3984 | 0.07598 |
| Citrate cycle (TCA cycle) | 20 | 0.27104 | 2 | 0.028597 | 3.5544 | 1 | 0.46328 | 0.09164 |
| Galactose metabolism | 26 | 0.35235 | 2 | 0.046613 | 3.0659 | 1 | 0.54917 | 0.03644 |
| Phenylalanine, tyrosine and tryptophan biosynthesis | 4 | 0.054208 | 1 | 0.053172 | 2.9342 | 1 | 0.54917 | 0.5 |
| Synthesis and degradation of ketone bodies | 5 | 0.06776 | 1 | 0.06604 | 2.7175 | 1 | 0.54917 | 0 |
| D-Glutamine and D-glutamate metabolism | 5 | 0.06776 | 1 | 0.06604 | 2.7175 | 1 | 0.54917 | 0 |
| Glycine, serine and threonine metabolism | 32 | 0.43367 | 2 | 0.067799 | 2.6912 | 1 | 0.54917 | 0.29197 |
| Cyanoamino acid metabolism | 6 | 0.081312 | 1 | 0.078743 | 2.5416 | 1 | 0.57983 | 0 |
| Nitrogen metabolism | 9 | 0.12197 | 1 | 0.11588 | 2.1552 | 1 | 0.72199 | 0 |
| Phenylalanine metabolism | 9 | 0.12197 | 1 | 0.11588 | 2.1552 | 1 | 0.72199 | 0.40741 |
| Valine, leucine and isoleucine biosynthesis | 11 | 0.14907 | 1 | 0.13984 | 1.9673 | 1 | 0.80906 | 0.33333 |
| Histidine metabolism | 15 | 0.20328 | 1 | 0.18593 | 1.6824 | 1 | 1 | 0 |
| Glycerolipid metabolism | 18 | 0.24394 | 1 | 0.21895 | 1.5189 | 1 | 1 | 0.28098 |
| Butanoate metabolism | 20 | 0.27104 | 1 | 0.24026 | 1.426 | 1 | 1 | 0 |
| Starch and sucrose metabolism | 23 | 0.3117 | 1 | 0.27118 | 1.305 | 1 | 1 | 0.03778 |
| Glutathione metabolism | 26 | 0.35235 | 1 | 0.30092 | 1.2009 | 1 | 1 | 0.00573 |
| Porphyrin and chlorophyll metabolism | 27 | 0.36591 | 1 | 0.31057 | 1.1693 | 1 | 1 | 0 |
|  | Total | Expected | Hits | Raw p | =-LOG(p) | Holm adjust | FDR | Impact |
| Cysteine and methionine metabolism | 28 | 0.37946 | 1 | 0.3201 | 1.1391 | 1 | 1 | 0.09464 |
| Valine, leucine and isoleucine degradation | 38 | 0.51498 | 1 | 0.40875 | 0.89465 | 1 | 1 | 0 |
| Primary bile acid biosynthesis | 46 | 0.6234 | 1 | 0.47167 | 0.75148 | 1 | 1 | 0.02976 |
